# The Effect of Adipose-Derived Stem Cell (ADSC)-Exos on the Healing of Autologous Skin Grafts in Miniature Pigs

**DOI:** 10.3390/ijms26020479

**Published:** 2025-01-08

**Authors:** Pujun Li, Lei Cao, Tao Liu, Xiangyu Lu, Yajun Ma, Hongbin Wang

**Affiliations:** 1 College of Veterinary Medicine, Northeast Agricultural University, Harbin 150300, China; pujunli1024@163.com (P.L.); caolei_001010@163.com (L.C.); liutaotiger@163.com (T.L.); lxy1997202206@163.com (X.L.); mayajun1994@126.com (Y.M.); 2 Heilongjiang Provincial Key Laboratory of Pathogenic Mechanism for Animal Disease and Comparative Medicine, Harbin 150300, China

**Keywords:** miniature pigs, adipose-derived mesenchymal stem cell exosomes, autologous skin grafting

## Abstract

The skin functions as the body’s primary defense barrier; when compromised, it can lead to dehydration, infection, shock, or potentially life-threatening conditions. Miniature pigs exhibit skin characteristics and healing processes highly analogous to humans. Mesenchymal stem cells contribute to skin injury repair through a paracrine mechanism involving exosomes. This research examines whether adipose-derived MSC exosomes effectively enhance healing following autologous skin grafting in miniature pigs. It also compares the roles and distinctions of ADSCs and ADSC-Exos in inflammatory responses and tissue regeneration. This study found significantly reduced levels of oxidative stress products and pro-inflammatory factors, while antioxidant factors, anti-inflammatory factors, and pro-regenerative factors were elevated, and anti-regenerative factor levels decreased. Moreover, the expression levels of key markers—namely, PI3K, Akt, and mTOR—in the regeneration-associated signaling pathway were increased. The alterations in these indicators indicate that ADSC-Exos can regulate inflammatory responses and promote regeneration. This study provides a novel theoretical foundation for the implementation of acellular therapy in clinical settings.

## 1. Introduction

The skin, as the body’s largest organ, covers the surface and directly interfaces with the external environment, serving as the primary defense against external threats [1]. When the skin sustains damage, its protective barrier is compromised, diminishing its ability to ward off external threats. Consequently, significant fluid loss may occur, potentially leading to symptoms such as dehydration, infection, and shock, which can pose a threat to survival [2]. Skin injury repair is a complex process involving the coordinated interaction of various cells, cytokines, and extracellular matrices. The healing process comprises four overlapping stages: coagulation, inflammation, proliferation, and remodeling [3]. Disruptions in any of these stages can result in chronic wounds and abnormal scar formation [4]. Autologous skin grafting is frequently employed to treat extensive areas of skin loss due to its higher survival rate and ability to rapidly close wounds, thereby reducing infection risk [5]. However, the long-term consequences of transplantation can be problematic, potentially leading to issues such as secondary scarring, contractures, necrosis, structural deformation, and poor wear resistance [6].

Mesenchymal stem cells (MSCs) have emerged as a prominent cell type in recent cell therapy research. Their widespread application in various disease studies is attributed to advantages such as ethical compliance, low immunogenicity, and effective therapeutic outcomes, leading to significant advancements [7,8,9]. Moreover, their capacity to suppress immune rejection, attenuate inflammatory responses, mitigate tissue fibrosis, and promote cell regeneration has drawn considerable attention in skin injury research [10]. However, challenges persist, including the potential for abnormal differentiation of MSCs, and the stringent requirements for storage and transportation. Extensive investigations into the paracrine signaling mechanisms of MSCs have revealed that exosomes derived from these cells (MSC-Exo) function as the primary bioactive agents mediating their therapeutic effects [11]. These exosomes contain bioactive constituents originating from mesenchymal stem cells and demonstrate effects that closely parallel those of the stem cells themselves [12]. Notably, MSC-Exos lack the ability to differentiate, positioning them as a promising candidate for acellular therapies. Studies indicate that both MSCs and MSC-Exo exhibit anti-inflammatory properties and promote healing in skin injuries among rodent and canine models [13,14]. However, significant anatomical and physiological differences exist between the skin of these animals and human skin. In contrast, the skin characteristics and healing processes observed in miniature pigs demonstrate greater similarity to those in humans, rendering them particularly suitable as experimental models for comparative medicine [15].

Our previous research has demonstrated that adipose-derived stem cells (ADSCs) significantly reduce inflammation following skin graft procedures in miniature pigs, thereby promoting skin healing and vascular regeneration [16]. However, the specific mechanisms by which exosomes derived from ADSCs (ADSC-Exos) contribute to reducing inflammation and enhancing wound healing during autologous skin grafting in miniature pigs remain to be elucidated. This study aims to establish a model for autologous skin transplantation in miniature pigs, utilizing ADSC-Exos as an interventional approach. The primary objective of this investigation is to evaluate the therapeutic efficacy of ADSC-Exos in this autologous skin transplantation model. Furthermore, we will analyze and compare the effects and underlying mechanisms of both ADSCs and their derived exosomes.

## 2. Results

### 2.1. ADSC-Exos Promote Skin Wound Healing

**Figure 1:**
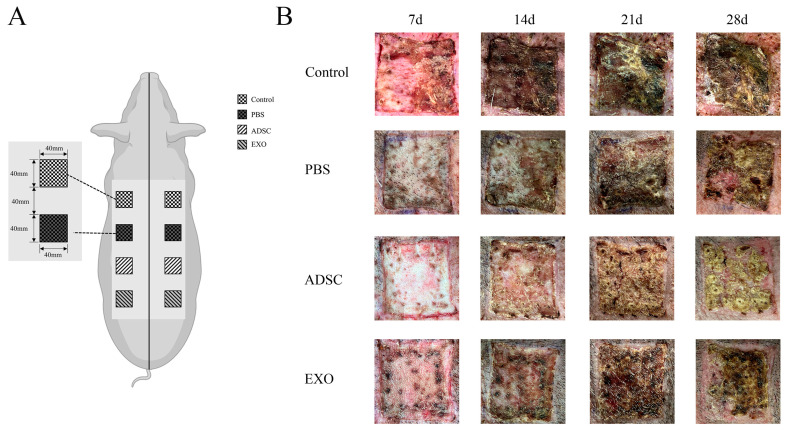
Model diagram and clinical observation results: (**A**) schematic diagram of a small pig autologous skin graft model; (**B**) clinical observation results of autologous skin grafting.

During the experiments, no infections or re-injuries occurred at the autologous skin graft sites in the miniature pigs. The animals maintained good mental status, with normal diet, exercise, and bowel movements. Clinical observations of the skin healing process were conducted. As illustrated in Figure 1B, by the 7th day post-surgery, all four groups of transplanted skin grafts had established blood supply with the original transplant site, demonstrating tight edge integration. The ADSC group and ADSC-Exo group exhibited a more reddish appearance compared to the Control group and PBS group. On the 14th day post-surgery, the transplanted skin grafts in the Control and PBS groups were dry, hard, and dark brown. In contrast, the grafts in the ADSC and ADSC-Exo groups were dry but reddish, with hair growth beginning in all four groups. By the 21st day post-operation, the edges of the transplanted skin grafts in the Control and PBS groups showed significant healing and scarring. The graft surfaces were covered by necrotic epidermal tissue, indicating the initiation of re-epithelialization. In the ADSC group and ADSC-Exo group, scabs began to detach from the wound edges. Notable hair growth was observed in all four groups of skin grafts. At 28 days post-surgery, the ADSC group and the ADSC-Exo group demonstrated extensive shedding of surface scabs, with the skin color approaching normal, albeit lacking elasticity compared to normal skin. The model group and PBS group began shedding surface scabs, with some transplanted skin pieces completing re-epithelialization and appearing dark red. Significant hair growth was observed in all four groups of skin grafts.

### 2.2. ADSC-Exos Improve Histopathological Changes After Skin Grafting

**Figure 2:**
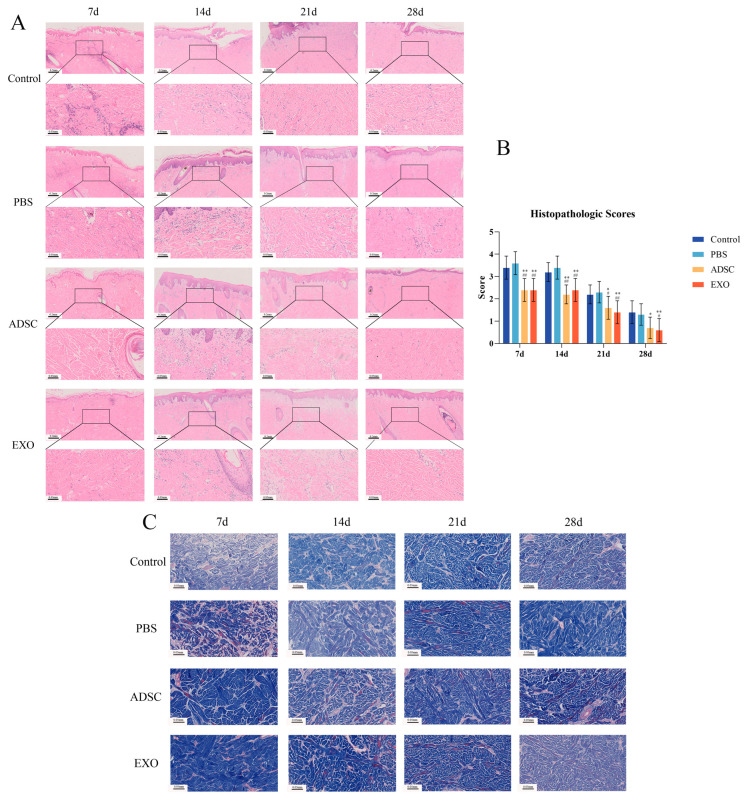
(**A**) HE staining results of skin tissue; (**B**) histopathological score results; and (**C**) MASSON staining results of skin tissue. ** *p* < 0.01 and * 0.01 < *p* < 0.05, compared to the Control group. ## *p* < 0.01 and # 0.01 < *p* < 0.05, compared to the PBS group.

Histopathological alterations in skin tissue were examined through HE and MASSON staining, as depicted in Figure 2A,C. Seven days post-surgery, both the Control and PBS groups exhibited significant infiltration of inflammatory cells, delayed vascularization, and epidermal layer necrosis. The ADSC group and the ADSC-Exo group demonstrated mild inflammatory cell infiltration and favorable growth status. In all four groups, specialized skin structures such as hair follicles, sebaceous glands, and sweat glands were either abnormal or absent. Furthermore, the newly formed collagen fibers displayed irregular arrangement.

Fourteen days after surgery, the Control and PBS groups continued to exhibit significant inflammatory cell infiltration, albeit reduced compared to day 7. Stratification of skin tissue and necrotic tissue was apparent. In the ADSC and ADSC-Exo groups, specialized skin structures progressively resembled normal conditions, displaying a more organized arrangement, and necrotic tissue began to slough off. Fibroblasts and collagen fibers proliferated markedly. All groups demonstrated thickening of the stratum corneum and increased collagen fiber content. The ADSC and ADSC-Exo groups showed higher collagen fiber density compared to the Control and PBS groups, although the arrangement remained disorganized.

Twenty-one days after surgery, the Control and PBS groups exhibited a reduction in inflammatory cell infiltration, accompanied by the proliferation of fibroblasts and collagen fibers, and the shedding of some necrotic epidermal tissue. The ADSC and ADSC-Exo groups demonstrated a higher number of fibroblasts and collagen fibers compared to the other two groups, with a more organized arrangement and denser structure. In the ADSC and ADSC-Exo groups, newly formed collagen fibers displayed uniform and regular deposition. Conversely, the Control and PBS groups showed less collagen generation, characterized by irregular and curved arrangements.

Twenty-eight days after surgery, the Control group and the PBS group exhibited dry necrotic epidermis shedding, with the surface covered by extensive proliferative tissue. Some transplanted skin pieces in these groups lacked an epidermal layer. In contrast, the ADSC and ADSC-Exo groups demonstrated complete skin tissue structure, characterized by a substantial amount of newly formed collagen fibers between the epidermis and dermis. Notably, in the ADSC and ADSC-Exo groups, collagen fibers transitioned from thick to thin, indicating a return to normal skin structure. Conversely, the Control group and the PBS group displayed extensive disordered collagen fiber deposition.

The results of the histopathological scoring are consistent with the microscopic observations, as shown in Figure 2B, presenting a gradually decreasing trend. The histopathological scores of the ADSC and ADSC-Exo groups were significantly lower than those of the Control and PBS groups (*p* < 0.05).

### 2.3. ADSC-Exos Inhibit Oxidative Stress After Skin Grafting

**Figure 3:**
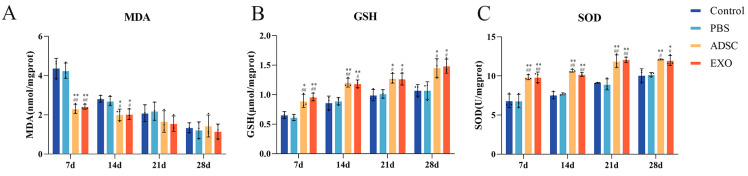
The effects of ADSC-Exos on oxidative stress. (**A**) Biochemical assay results show the levels of MDA in skin tissue; (**B**) biochemical assay results show the levels of GSH in skin tissue; and (**C**) biochemical assay results show the levels of SOD in skin tissue. ** *p* < 0.01 and * 0.01 < *p* < 0.05, compared to the Control group. ## *p* < 0.01 and # 0.01 < *p* < 0.05, compared to the PBS group.

Figure 3 demonstrates that, following skin grafting, the levels of the antioxidant GSH and SOD activity in the Control, PBS, ADSC, and ADSC-Exo groups increased progressively over time, peaking at 28 days. At all four time points, the ADSC and ADSC-Exo groups exhibited significantly higher SOD activity and GSH levels compared to the Control and PBS groups (*p* < 0.05). Moreover, the levels of the oxidative stress product MDA decreased gradually in all groups as healing advanced. At 7 and 14 days post-surgery, the MDA levels in the ADSC and ADSC-Exo groups were significantly lower than those in the Control and PBS groups (*p* < 0.05). At 21 and 28 days post-surgery, the MDA levels stabilized, revealing no significant differences among the groups (*p* > 0.05).

### 2.4. ADSC-Exos Regulate Inflammatory Balance After Skin Grafting

**Figure 4:**
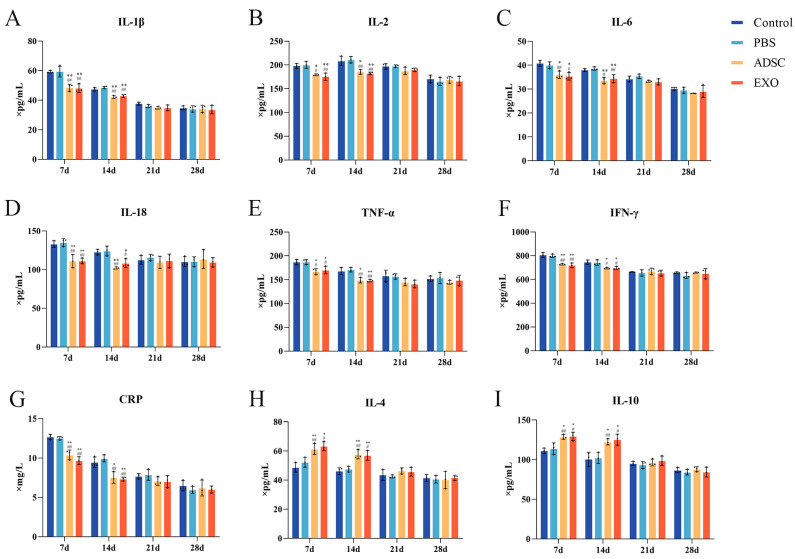
(**A**–**I**) Elisa results show the levels of IL-1β, IL-2, IL-6, IL-18, TNF-α, CRP, IFN-γ, IL-4, and IL-10 in skin tissue. ** *p* < 0.01 and * 0.01 < *p* < 0.05, compared to the Control group. ## *p* < 0.01 and # 0.01 < *p* < 0.05, compared to the PBS group.

**Figure 5:**
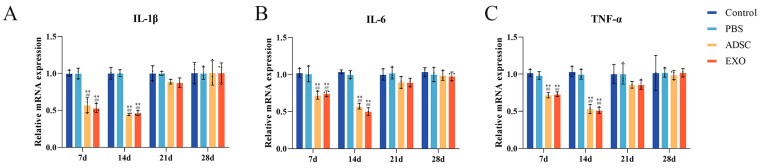
(**A**–**C**) RT-qPCR results show the mRNA levels of IL-1β, IL-6, and TNF-α. ** *p* < 0.01, compared to the Control group. ## *p* < 0.01, compared to the PBS group.

Following skin grafting, the levels of pro-inflammatory cytokines, including IL-1β, IL-2, IL-6, IL-18, TNF-α, CRP, and IFN-γ, exhibited a gradual decrease over time in the Control, PBS, ADSC, and ADSC-Exo groups. At 7 and 14 days post-surgery, the levels of each factor in the ADSC and ADSC-Exo groups were significantly lower compared to the Control and PBS groups (*p* < 0.05). By 21 and 28 days post-surgery, the levels of each factor stabilized, with no significant differences observed between the groups (*p* > 0.05). The levels of anti-inflammatory factors IL-4 and IL-10 in the tissue initially increased and subsequently decreased as healing progressed. At 7 and 14 days post-surgery, the levels of IL-4 and IL-10 in the ADSC and ADSC-Exo groups were significantly higher than those in the Control and PBS groups (*p* < 0.05). At 21 and 28 days post-surgery, the levels of IL-4 and IL-10 stabilized, with no significant differences between the groups (*p* > 0.05). The mRNA expression of IL-1β, IL-6, and TNF-α in the tissue corresponded with the changes in their Elisa levels (Figure 4 and Figure 5).

### 2.5. ADSC-Exos Promote Regeneration After Skin Grafting

**Figure 6:**
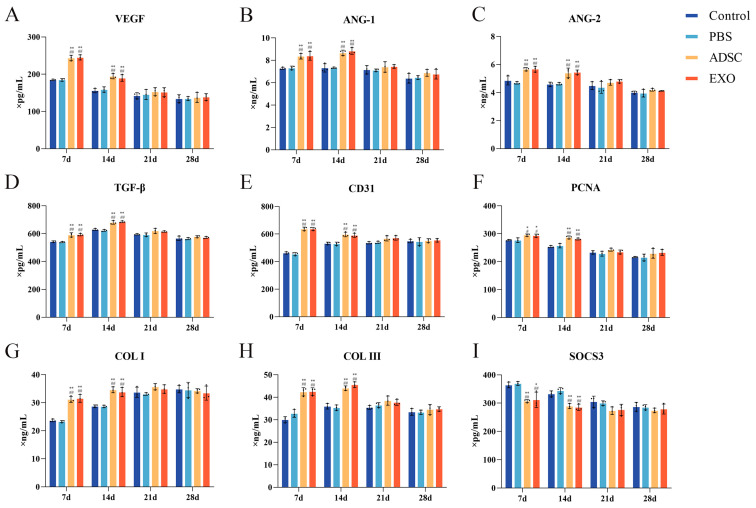
(**A**–**I**) Elisa results show the levels of VEGF, ANG-1, ANG-2, CD31, TGF-β, PCNA, COL I, COL III, and SOCS3 in skin tissue. ** *p* < 0.01 and * 0.01 < *p* < 0.05, compared to the Control group. ## *p* < 0.01 and # 0.01 < *p* < 0.05, compared to the PBS group.

**Figure 7:**
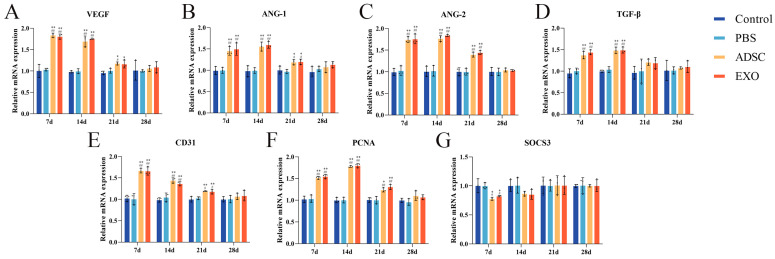
(**A**–**G**) RT-qPCR results show the mRNA levels of VEGF, ANG-1, ANG-2, CD31, TGF-β, PCNA, and SOCS3. ** *p* < 0.01 and * 0.01 < *p* < 0.05, compared to the Control group. ## *p* < 0.01 and # 0.01 < *p* < 0.05, compared to the PBS group.

**Figure 8:**
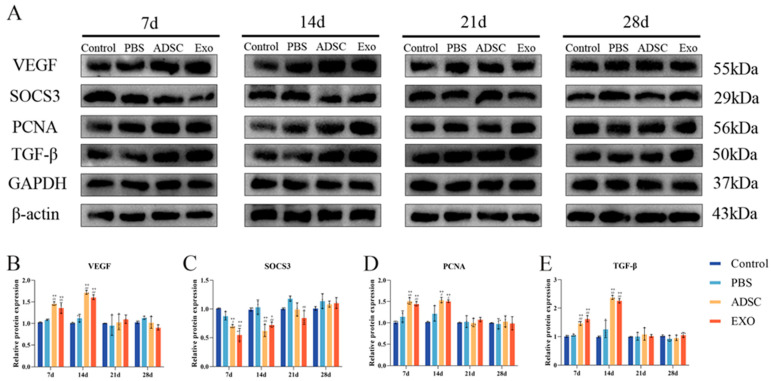
(**A**–**E**) Representative protein blot analysis and quantification of VEGF, TGF-β, PCNA, and SOCS3. ** *p* < 0.01 and * 0.01 < *p* < 0.05, compared to the Control group. ## *p* < 0.01 and # 0.01 < *p* < 0.05, compared to the PBS group.

**Figure 9:**
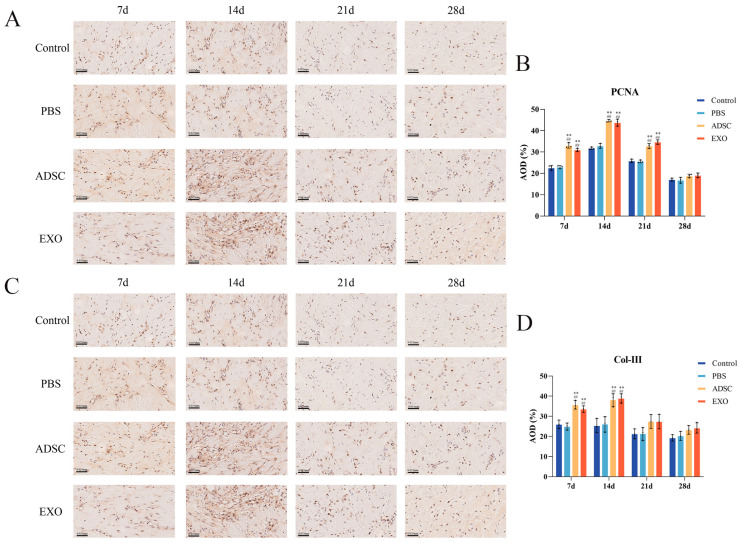
(**A**,**B**) PCNA immunohistochemistry results and analysis. (**C**,**D**) COL III immunohistochemistry results and analysis. ** *p* < 0.01, compared to the Control group. ## *p* < 0.01, compared to the PBS group.

To further investigate skin tissue regeneration and healing, we measured the levels of key factors (Figure 6, Figure 7, Figure 8 and Figure 9). The levels of VEGF, ANG-1, ANG-2, and CD31, which are related to endothelial cell regeneration, were significantly upregulated after skin grafting. On post-operative days 7 and 14, the levels of VEGF, ANG-1, ANG-2, and CD31 in the ADSC and ADSC-Exo groups were significantly higher than those in the Control and PBS groups (*p* < 0.05). By post-operative day 21, the levels gradually stabilized, with no significant differences between the groups (*p* > 0.05). Similarly, the levels of PCNA, TGF-β, Collagen I, and Collagen III, which are associated with cell proliferation and differentiation, were also significantly upregulated after skin grafting. At 7 and 14 days post-surgery, the levels of PCNA, TGF-β, Collagen I, and Collagen III in the ADSC and ADSC-Exo groups were significantly higher than those in the Control and PBS groups (*p* < 0.05). By post-operative day 21, the levels stabilized, showing no significant differences between the groups (*p* > 0.05). The results for the negative regulatory factor SOCS3 indicated that, at post-operative days 7 and 14, levels in the ADSC and ADSC-Exo groups significantly decreased compared to the Control and PBS groups *p* < 0.05), before gradually stabilizing. Additionally, we also measured the mRNA expression levels of VEGF, ANG-1, ANG-2, CD31, TGF-β, SOCS3, and PCNA in the skin tissue, as well as the protein expression levels of VEGF, PCNA, TGF-β, and SOCS3. These results were generally consistent with those obtained from Elisa.

### 2.6. ADSC-Exos Activate the PI3K/AKT/mTOR Signaling Pathway

**Figure 10:**
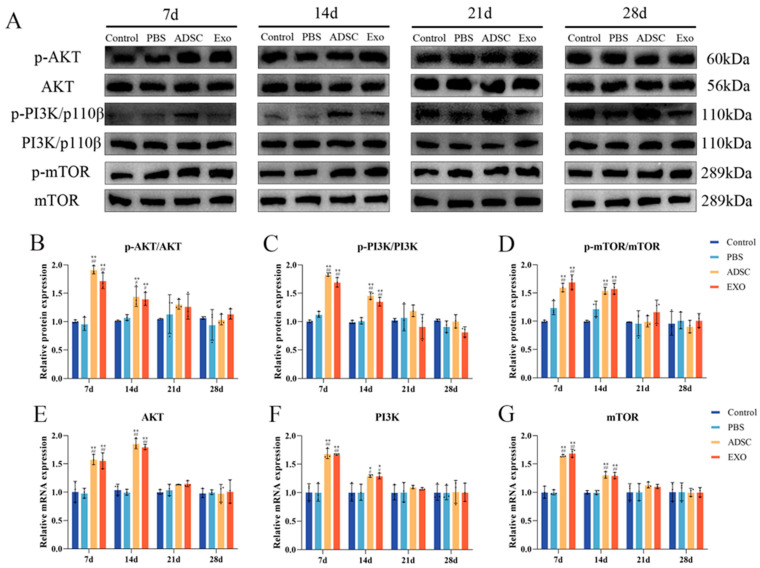
Results related to factors in the PI3K/AKT/mTOR signaling pathway. (**A**–**D**) Representative Western blot analysis and quantification of p-Akt, Akt, p-PI3K/p110β, PI3K/p110β, p-mTOR, and mTOR. (**E**–**G**) RT-qPCR results showing the mRNA levels of Akt, PI3K, and mTOR. ** *p* < 0.01 and * 0.01 < *p* < 0.05, compared to the Control group. ## *p* < 0.01 and # 0.01 < *p* < 0.05, compared to the PBS group.

To determine whether ADSC-Exos activate the AKT/PI3K/mTOR pathway for regeneration, the expression of AKT, PI3K, and mTOR proteins and genes was analyzed. Figure 10 demonstrates that the relative expression levels of AKT, PI3K, and mTOR increased significantly following skin transplantation. At 7 and 14 days post-surgery, the levels of AKT, PI3K, and mTOR in the ADSC group and ADSC-Exo group were markedly higher than those in the Control group and PBS group (*p* < 0.05). By 21 days post-surgery, the levels stabilized, showing no significant differences between the groups (*p* > 0.05). The mRNA levels of AKT, PI3K, and mTOR exhibited a correlation with their respective protein expression levels.

### 2.7. ADSC-Exos Have a Similar Regenerative Ability to ADSCs After Skin Transplantation

Seven days after surgery, the skin structure, histopathological changes, inflammatory infiltration, and collagen fiber content were comparable in both the ADSC and the ADSC-Exo groups. Only on the 14th day post-surgery, the ADSC-Exo group exhibited slightly less inflammatory infiltration compared to the ADSC group. By the 28th day post-surgery, cell density and collagen fiber recovery were similar in both intervention groups. Furthermore, the expression levels of inflammatory factors and regeneration-related factors in the skin tissues were comparable between the ADSC and the ADSC-Exo groups. Although each group demonstrated distinct advantages and disadvantages, no statistically significant differences were observed between the two groups (*p* > 0.05).

## 3. Discussion

Adipose-derived stem cells (ADSCs) have emerged as a prominent cell source in regenerative medicine due to their accessibility from adipose tissue, low immunogenicity, and multi-lineage differentiation potential. Since their initial isolation from liposuction-derived adipose tissue by Zuk et al. [17] in 2001, ADSCs have demonstrated promising clinical applications across various fields [18,19,20]. Research indicates that ADSCs mediate the therapeutic effects of mesenchymal stem cells on diverse diseases through paracrine mechanisms, involving factors such as cytokines, exosomes, and nucleic acids. ADSCs secrete bioactive factors that stimulate fibroblast proliferation at injury sites, enhance angiogenesis, and exhibit anti-inflammatory properties, collectively accelerating wound healing [21,22,23]. Jin et al. [24] reported that ADSCs promote skin regeneration and angiogenesis in pressure ulcer patients by activating the PPARβ/δ pathway through paracrine signaling. Li et al. [25] demonstrated through in vitro experiments that ADSCs can accelerate angiogenesis and extracellular matrix (ECM) remodeling, thereby enhancing wound healing in diabetic mice. However, the clinical application of MSCs presents certain safety concerns, including immunocompatibility, tumorigenicity, and embolism risk [26,27]. Extracellular vesicles (EVs) produced by the paracrine secretion mechanism are roughly divided into three subgroups based on their biological origin, release pathways, size, content, and function [28]: apoptotic vesicles, 50–5000 nm; microvesicles, 100–1000 nm; and exosomes, 30–200 nm [29]. The overlap in size ranges indicates the limitations of this classification [30]. Our previous results showed that the isolated vesicles have a typical bilayer structure with uniform cup-shaped characteristics and express high levels of exosome-specific markers, including CD 63, CD 81, and TSG 101. Additionally, NTA results indicated a uniform particle size distribution, with 98.9% of the vesicles measuring approximately 121.6 nm in diameter [31]. Therefore, we identified these vesicles as ADSC-Exos. Recent studies have revealed that exosomes are a key component of the therapeutic effects exerted by ADSCs through paracrine mechanisms [32]. Consequently, stem cell exosomes have attracted significant attention in wound healing therapy, as they can mitigate issues associated with live cells, such as immunocompatibility, tumor development, and genetic diversity [33]. Exosomes facilitate intercellular communication by transferring substances to recipient cells, thereby promoting the growth and proliferation of skin cells involved in wound healing [34,35,36].

Skin wound healing encompasses intricate and dynamic stages of tissue regeneration, necessitating the coordinated efforts of immune cells, hematopoietic cells, and resident skin cells [37]. The restoration of dermal skin wounds involves four interconnected phases: hemostasis, inflammation, proliferation, and remodeling. Each phase comprises several meticulously regulated sub-stages. These phases are interdependent and operate in a continuous, overlapping manner [4].

Following skin damage, the body rapidly initiates a response by aggregating platelets and fibrinogen to form a temporary blood clot, effectively sealing the wound [38]. In the ensuing hours, an inflammatory response occurs, characterized by local congestion, serous exudation, and white blood cell migration, resulting in redness and swelling at the wound site. During this phase, platelet-released cytokines attract neutrophils and macrophages to the injury site, which, alongside resident mast cells, begin to eliminate invading microorganisms and clear cellular debris from the wound area [39]. However, excessive inflammatory responses can impede the wound healing process and potentially lead to the development of chronic, non-healing wounds. Therefore, modulating the inflammatory response during wound healing is a crucial aspect of skin wound repair. Studies have shown that reducing the inflammatory response of wounds can mitigate scar formation [40,41]. The mechanisms through which exosomes attenuate inflammatory responses are diverse and involve multiple pathways [42,43]. Notably, ADSC-Exos play a prominent role in reducing inflammation [44]. Zhao et al. [45] found that ADSC exosomes can direct macrophages to adopt the M2 phenotype, thereby reducing their capacity to activate inflammatory responses. Furthermore, research by Li et al. [46] indicated that ADSC-Exos with high expression of Nrf2 can decrease the levels of inflammatory cytokines such as IL-1β, IL-6, and TNF-α, thus diminishing the inflammatory response in wounds. Our study demonstrates that ADSC-Exos not only reduced the levels of pro-inflammatory factors, including IL-1β, IL-2, IL-6, IL-18, TNF-α, CRP, and IFN-γ, but also significantly increased the levels of anti-inflammatory factors IL-4 and IL-10. This regulation contributes to restoring the inflammatory balance at the injury site and reducing inflammatory infiltration.

Local blood circulation plays a crucial role in wound healing [47]. A robust blood supply provides essential nutrients and oxygen for tissue regeneration, promotes cell proliferation and granulation tissue formation, and facilitates the absorption of necrotic tissue while controlling local infections [48]. Studies have demonstrated that ADSC-Exos can enhance endothelial cell proliferation, migration, and angiogenesis [49]. This enhancement increases local blood circulation and expedites the wound healing process. The improvement in angiogenesis is primarily attributed to the high expression of specific microRNAs [50]. ADSC-Exos contain miRNAs (miR-126, miR-130a, miR-132, and miR-378a-3P) [51,52] that not only stimulate the proliferation and migration of endothelial cells but also promote angiogenesis by elevating the levels of various growth factors such as VEGF, EGF, and FGF in vascular endothelial cells [52]. Additional research has revealed that ADSC-Exos can enhance endothelial cell migration and blood vessel formation by transporting functional enzymes, including matrix metalloproteinases (MMPs), thereby accelerating the process of angiogenesis [53]. Our findings indicate that ADSC-Exos significantly elevate the expression of factors such as VEGF, ANG-1, ANG-2, TGF-β, and CD31, expediting angiogenesis and promoting tissue regeneration.

Promoting fibroblast proliferation and migration is essential for effective skin wound healing and contraction. Fibroblasts play a crucial role in fibrin clot degradation, ECM remodeling, and collagen fiber formation [54]. ADSC-Exos can enhance the proliferation and migration of fibroblasts through various mechanisms. Several studies have shown that miRNA derived from ADSC-Exos positively influences fibroblast function. Recent research has extensively explored how ADSC-Exos affect fibroblast proliferation and migration. Evidence suggests that ADSC-Exos are instrumental in forming corneal stromal fibroblasts, enhancing their vitality, and facilitating the transformation of corneal stromal cells into fibroblasts [55]. Furthermore, ADSC-Exos can promote skin wound healing by optimizing fibroblast characteristics. Fibroblasts stimulated by ADSC-Exos exhibit a dose-dependent increase in cell migration, proliferation, and collagen synthesis. This results in elevated levels of Collagen I, Collagen III, MMP-1, bFGF, and TGF-β, significantly enhancing wound healing in vivo [56]. In vitro experiments have demonstrated that ADSC-Exos can increase the levels of phosphorylated Akt (p-Akt), a crucial signaling pathway for promoting wound healing [57]. Corroborating this view, Ren et al. suggest that ADSC-Exos accelerate the activation of both AKT and ERK signaling pathways in fibroblasts, thereby promoting their proliferation and migration in vitro and in vivo [58]. Our research indicates that ADSC-Exos significantly upregulate the levels of related factors PCNA, COL I, and COL III, while significantly downregulating the level of SOCS3. This may promote cell proliferation and migration by regulating the PI3K/AKT/mTOR signaling pathway, accelerating tissue regeneration and wound healing.

The comparative analysis of ADSC-Exos and ADSCs on autologous skin graft model healing reveals that both treatments mitigate skin cell damage and enhance regeneration, with no statistically significant differences observed between them. Both ADSC-Exos and ADSCs demonstrate a protective effect on skin autografts. Consequently, as cytokines secreted by ADSCs, ADSC-Exos may play a primary role in the application of ADSCs for skin autograft treatment. This study underscores the substantial efficacy of ADSC-Exos in skin autografts. Considering its safety profile and ease of processing as an acellular therapeutic product [59,60], ADSC-Exos shows enormous potential in clinical applications. Therefore, further investigation into the protective mechanisms of ADSC-Exos in autologous skin grafting is crucial for their clinical advancement.

This study presents certain limitations, namely that it only observes and detects up to 28 days post-surgery. Skin wound healing is a complex dynamic process that includes four overlapping stages: hemostasis, inflammation, proliferation, and remodeling. However, depending on the severity of the trauma and other factors, the duration of the remodeling phase of skin wound healing can range from twenty-one days to a year [61]. Clinical observations indicate that at 28 days post-surgery, the surface of the transplanted skin in each group still has varying degrees of scab coverage, and the specific time of scab shedding remains unknown. Furthermore, the levels of factors in the skin tissues of each group beyond 28 days are still unknown. Therefore, the specific mechanism of action of ADSC-Exos during the remodeling phase still requires further research.

## 4. Materials and Methods

### 4.1. Animals

Twelve healthy miniature pigs, with six males and six females, were maintained in a standard environment, aged four to five months, and weighing 20 to 30 kg, with unrestricted access to food and water. All experimental procedures received approval from the Animal Ethics Committee at Northeast Agricultural University.

### 4.2. Acquisition of ADSC and ADSC-Exos

ADSCs and ADSC-Exos were isolated using previously described methods [31]. In brief, the following procedure was used: Anesthetize small pigs. Remove the fat from the groin area, including the fascia and blood vessels. Cut the fat into 1 mm^3^ fragments. Digest the fragments in a water bath at 37 °C using 0.1% type I collagenase (BioFroxx, Guangzhou, China). Then, centrifuge the mixture at 300× *g* for 10 min. Resuspend the lower precipitate in a complete culture medium (Gibco, Boston, MA, USA), then filter it through a 70 μm filter (Merck, Darmstadt, Germany). Continue centrifuging under the same conditions, resuspend in a complete culture medium, and inoculate into culture flasks at a density of 3 × 10^4^. After 24 h, change the culture medium. Subculture the cells when they reach 70–80% confluence When the 4th–5th generation cells grow to 80%, remove the complete culture medium, wash with PBS (BioShark, Hefei, China), and replace with serum-free medium for 36 h, then collect the supernatant. Centrifuge the mixture in three steps, first at 300× *g* for 15 min, then at 2000× *g* for 25 min, and finally at 10,000× *g* for 50 min, to remove live cells, dead cells, and cell debris. Subsequently, filter the clarified supernatant through a 0.22 μm filter to remove larger extracellular vesicles, and then concentrate the supernatant 10 times by centrifuging through a 100 kDa ultrafiltration tube (Merck, Darmstadt, Germany). After ultracentrifuging at 100,000× *g* for 90 min (Beckman, Brea, CA, USA), resuspend the precipitate in PBS, and ultracentrifuge again at 100,000× *g* for 90 min to remove the medium and soluble proteins. Finally, resuspend the precipitate composed of ADSC-Exos in PBS and store it at −80 °C.

### 4.3. Surgical Procedure and Treatment Protocol

Preoperative procedures include isolating the pig, fasting for 24 h, restricting water intake for 12 h, weighing, and shaving the back hair from the shoulder blade to the hip joint. They also include establishing venous access at the ear margin, inducing anesthesia with propofol (Libang, Xian, China), intubating the pig, positioning it in a prone restraint, maintaining anesthesia using isoflurane (RWD, Shenzhen, China), and adjusting the operating table temperature to approximately 37 °C. Routine disinfection of the back of the small pig is performed, and the surgical site is marked with a sterile marker. Four 16 cm^2^ square surgical sites are marked on each side of the midline of the back, 3 cm from the midline, with a 4 cm interval between each site. A second disinfection is performed, followed by draping with a surgical towel. The skin flap is removed, and subcutaneous tissue and part of the dermis are excised in saline solution (Medisan, Hrabin, China) for repositioning and transplantation, followed by nodular suturing (Figure 1A). During this stage, the skin is divided into four groups: one group remains untreated, one group receives a 2 mL injection of sterile PBS solution at four points in the fascia layer, another group is injected with 8 × 10^5^ ADSCs dissolved in 2 mL of sterile PBS solution at four points [16], and the last group is injected with 200 µg of ADSC-Exos in 2 mL of sterile PBS solution at four points. After the injection, the site is disinfected with iodine tincture, the transplant site is covered with petroleum jelly gauze, and sterile gauze is applied for pressure bandaging. After the operation, each pig is isolated and kept separately. The sutures and bandages are removed on the seventh day. On the 7th, 14th, 21st, and 28th days post-operation, three pigs are randomly selected to collect tissue samples from one side of their skin while photographing and documenting the healing condition on the opposite side (for the concentration screening of ADSC-Exos, please refer to Supplementary Materials).

### 4.4. Enzyme-Linked Immunosorbent Assay (Elisa)

According to the manufacturer’s instructions (MEIMIAN, Wuhan, China), specific Elisa kits were used to measure the levels of inflammation-related factors in skin tissue, including interleukin 1 beta (IL-1β), interleukin 2 (IL-2), interleukin 4 (IL-4), interleukin 6 (IL-6), interleukin 10 (IL-10), interleukin 18 (IL-18), tumor necrosis factor (TNF-α), C-reactive protein (CRP), and interferon-gamma (IFN-γ). Determination of the content of healing-related factors in skin tissue was achieved, including platelet–endothelial cell adhesion molecule (CD31), vascular endothelial growth factor (VEGF), transforming growth factor-β (TGF-β), angiopoietin-1 (ANG-1), angiopoietin-2 (ANG-2), proliferating cell nuclear antigen (PCNA), type I collagen (Collagen I), type III collagen (Collagen III), and suppressor of cytokine signaling 3 (SOCS3).

### 4.5. Analysis of Enzyme Activity Related to Oxidative Stress

The enzyme activity content in skin tissue was detected according to the instructions (NJJCbio, Nanjing, China) of the malondialdehyde (MDA), superoxide dismutase (SOD), and reduced glutathione (GSH) assay kits. The total protein content in the tissue was quantified using a protein assay kit (Beyotime, Shanghai, China), and the activity units were calculated.

### 4.6. Histopathological Assessment

A fresh skin tissue sample of 1 cm^3^ is taken. It is fixed in 4% paraformaldehyde (BioSharp, Hefei, China), embedded in paraffin, and cut into sections 4–5 μm thick. Hematoxylin and eosin (HE) staining and Masson’s trichrome (MASSON) staining are performed according to standard protocols. The histopathological changes were observed, and the tissue sections were scored according to the scoring criteria [62] (Table 1).

### 4.7. Real-Time Quantitative PCRwomen

According to the manufacturer’s instructions (Invitrogen, Carlsbad, CA, USA), total RNA was extracted from skin samples using a TRIzol reagent (Invitrogen, CA, USA). Following extraction, the RNA underwent reverse transcription to synthesize complementary DNA (Vazyme, Nanjing, China). The generated cDNA samples were subsequently tagged with SYBR Green I fluorescent dye (innovagene, Wuhan, China). The relative expression levels of the genes were quantified employing the 2^−ΔΔCt^ method, utilizing β-actin as the reference gene, following the manufacturer’s guidelines (Innovagene, Changsha, China) on the LightCycler 480 instrument (Roche, Basel, Switzerland). The primers used for this method were synthesized by UW Genetics and are detailed in Table 2.

### 4.8. Western Blotting

Skin samples were subjected to lysis utilizing a RIPA buffer (Beyotime, Shanghai, China) supplemented with 1% protease inhibitor (Beyotime, Shanghai, China) and 1% phosphatase inhibitor (Merck, Darmstadt, Germany). The protein concentration was quantified via the BCA assay (Beyotime, China) and subsequently normalized against a saline standard. Following this, the samples were denatured through boiling in a loading buffer (Beyotime, Shanghai, China) and then analyzed using SDS-PAGE (LEAGENE, Beijing, China). The protein bands were subsequently transferred to NC or PVDF membranes (Pall, Boston, MA, USA) employing a wet transfer technique. After the transfer, the membranes were blocked with either 5% skim milk (Wandashan, Harbin, China) or 5% BSA (BioSharp, Hefei, China) at room temperature for a duration of 2 h, incubated overnight at 4 °C with an anti-PCNA antibody (1:600, Proteintech, 60097-1-ig, Wuhan, China), TGF-β (1:1000, Immunoway, YT 4632, USA), SOCS 3 (1:1000, Uping, YP-Ab-03444, Hangzhou, China), VEGF (1:2000, Proteintech, 19003-1-AP, Wuhan, China), AKT (1:1000, Wanlei, WL0003b, Shenyang, China), p-AKT (1:1000, Wanlei, WLp001a, Shenyang, China), PI3K/p110β (1:1000, Wanlei, WL03380, Shenyang, China), p-PI3K/p110β (1:1000, Bioss, bs-6417R, Beijing, China), mTOR (1:1000, Abmart, T55306, Shanghai, China), p-mTOR (1:1000, Abmart, T56571, Shanghai, China), GAPDH (1:20,000, Proteintech, 10494-1-AP, Wuhan, China), and β-actin (1:40,000, Proteintech, 81115-1-RR, Wuhan, China). The next day, the membrane was incubated with the secondary antibody (1:10000,Proteintech, Wuhan, China) at room temperature for 2 h. ECL (meilunbio, Dalian, China) and the Tanon 5200 system were used to visualize the target protein bands, and their average optical densities were determined using ImageJ 1.52a (Java 1.8.0_112 64-bit).

### 4.9. Immunohistochemistry

Immunohistochemical methods were used to detect the expression levels of PCNA and Collagen III in skin tissue. Skin tissue specimens were cut to appropriate sizes, fixed in 4% paraformaldehyde for 24 h, embedded in paraffin, and sectioned. The sections were dewaxed overnight in an 80 °C oven, then immersed in a 3% H_2_O_2_ solution in the dark for 10 min to block endogenous peroxidase. Antigen retrieval was then performed using sodium citrate antigen retrieval solution(Beyotime, Shanghai, China) in a pressure cooker. Sections were sealed with BSA at room temperature for 20 min, followed by overnight incubation at 4 °C with primary antibodies PCNA (1:200, Proteintech, 60097-1-ig, Wuhan, China) and Collagen III (1:200, Uping, YP-Ab-17959, Hangzhou, China). Then, they were incubated at room temperature for 30 min with HRP labeled with streptavidin (Sigma, St. Louis, MO, USA). Ultimately, the specimens were subjected to staining with DAB (Proteintech, Wuhan, China) and hematoxylin (Leagene, Beijing, China), followed by sealing with neutral gum and drying in an oven. The stained sections from each group were examined microscopically, and analysis was performed using ImageJ 1.52a (Java 1.8.0_112 64-bit).

### 4.10. Statistical Analysis

Statistical analysis and charting were performed using GraphPad Prism 8.0.2 software. First, normality and homogeneity of variance analysis on the data were conducted. Once it meets the standards, a one-way ANOVA was used to analyze the differences between groups. After obtaining the results, we used Tukey HSD for post hoc testing to determine the final significant differences. A *p*-value of <0.01 indicates a highly significant statistical difference, 0.01< *p* < 0.05 indicates a significant statistical difference, and *p* > 0.05 indicates no significant statistical difference. Experimental data are presented as mean ± standard deviation (mean ± SD).

## 5. Conclusions

This study demonstrates that ADSC-Exos may inhibit inflammation following autologous skin grafting by upregulating the PI3K/Akt/mTOR signaling pathway, promoting tissue regeneration, thereby mitigating pathological damage and ultrastructural changes in the skin, and accelerating tissue healing. These findings provide a novel theoretical foundation for the implementation of acellular therapy in clinical settings.

## Figures and Tables

**Table 1:** Histopathological scoring criteria for the degree of skin injury after autologous skin transplantation.

Score	Inflammatory Infiltration	Epidermal Abnormalities and Necrosis	Cell Apoptosis and Necrosis
0	None or Minimal	None	None
1	Minimal	Minimal	None
2	Mild	Mild	Minimal
3	Moderate	Moderate	Moderate
4	Severe	Severe	Severe

**Table 2:** Gene-specific primers used for the RT-qPCR.

Gene	Forward Primer Sequence (5′-3′)	Reverse Primer Sequence (5′-3′)
VEGF	CATGGCAGAAGGAGACCAGAAACC	CACAGGACGGCTTGAAGATGTACTC
PCNA	GGCTCTATCCTGAAGAAGGTGCTG	GACATGAGACGAGTCCATGCTCTG
CD31	GGTGGTCAAGAGAAGCAATGAGGTC	AAATGGGCGAGGTTCCGTTTATGG
TGF-β	CCATTCGCGGCCAGATT	GCTCCGGTTCGACACTTTC
ANG-1	AAATGGAGGGGAAGCACAAGGAAG	ACTGTTATTGGTGGTGGCTCTGTTC
ANG-2	CACCTACACGCTGACCTTTCCTAAC	CGCTGAATAACTGTCCATCCACCTC
SOCS3	GGTCACCCACAGCAAGTTTCCC	TCCAGTAGAAGCCGCTCTCCTG
TNF-α	ACCAGCCAGGAGAGAGACAAG	AGCGTGTGAGAGGGAGAGAGT
IL-6	AGCAAGGAGGTACTGGCAGA	AAGACCGGTGGTGATTCTCA
IL-1β	GGGAGGATATCAAGGAGCACG	CTTGGAGCTTGCTAAAGGCAC
AKT	GACGGCACCTTCATCGGCTAC	CGCCACGGAGAAGTTGTTGAGG
PI3K	ACGGAGGAGGTGCTCTGGAAC	GGACTCGGGACTGGGCATCTC
mTOR	GCACGTCAGCACCATCAACCTC	GCCTCAGCCATTCCAACCAGTC
β-actin	TCTGGCACCACACCTTCT	TGATCTGGGTCATCTTCTCAC

## Data Availability

The data used to support the findings of this study are available from the corresponding author upon request.

## Supplementary Materials

The supporting information can be downloaded at https://www.mdpi.com/article/10.3390/ijms26020479/s1.
