# Supplementary material for: The Effect of Adipose-Derived Stem Cell (ADSC)-Exos on the Healing of Autologous Skin Grafts in Miniature Pigs
Source: Int J Mol Sci. 2025 Jan 8;26(2):479. doi: 10.3390/ijms26020479 (PMC11764972; doi:10.3390/ijms26020479)
Supplement: Supplementary file 1 [file ijms-26-00479-s001.zip › ijms-3288771-supplementary.pdf]

AKT

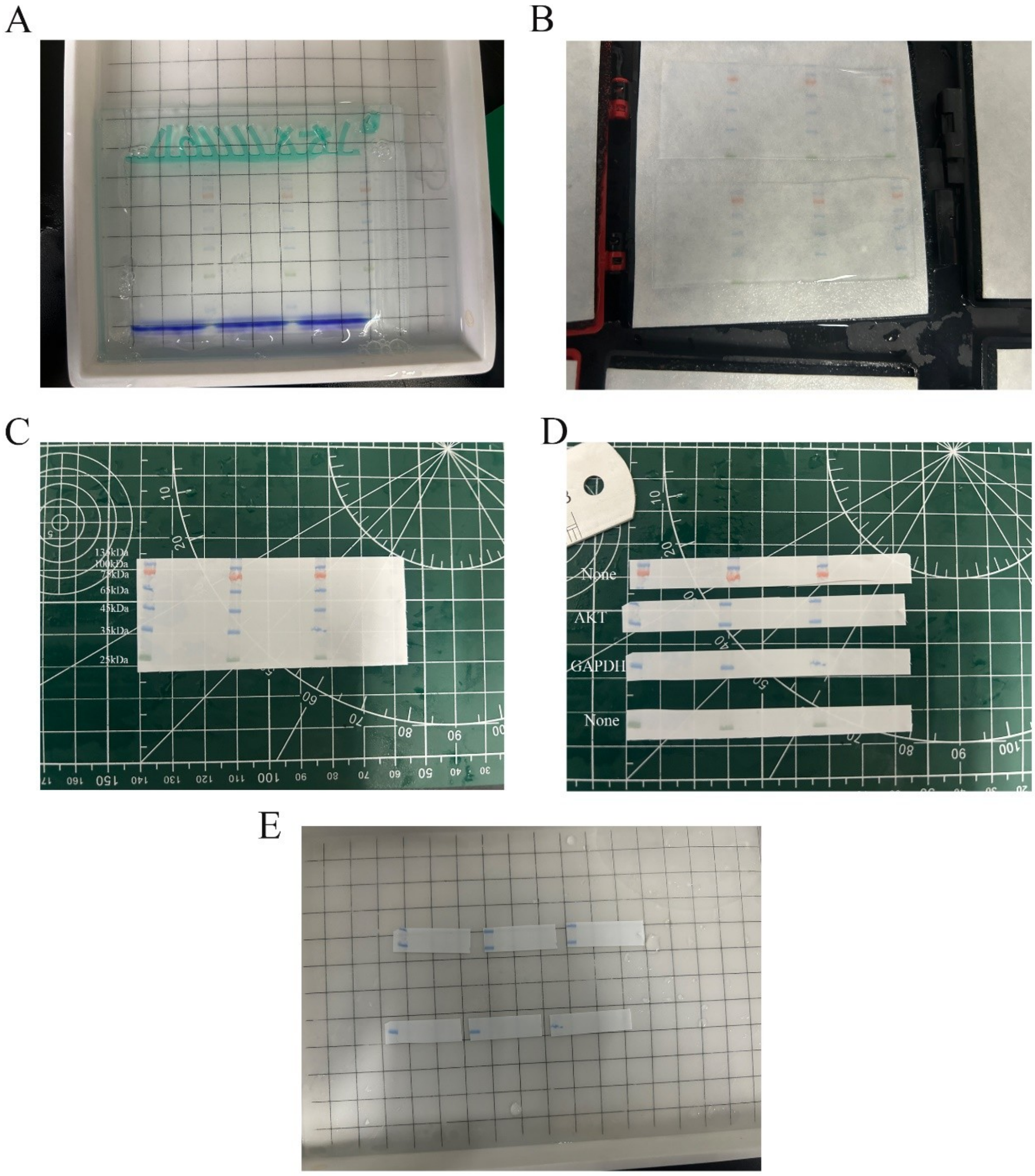

Western blotting flowchart.

A: Gel electrophoresis completed; B: Excess gel removed; C: Membrane transfer completed;

D: Incubation of strips with antibodies; E: Strip exposure.

mTOR

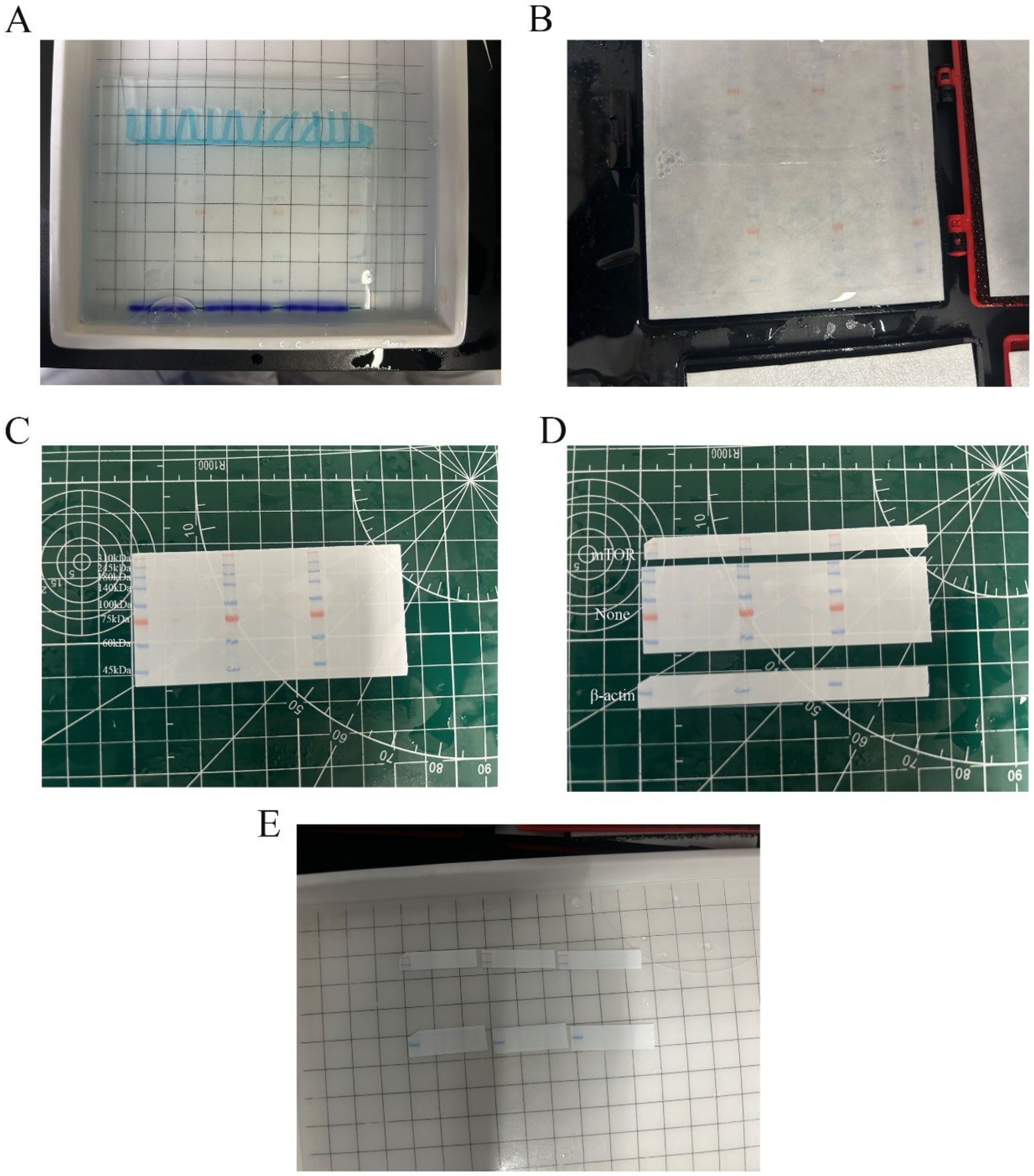

Western blotting flowchart.

A: Gel electrophoresis completed; B: Excess gel removed; C: Membrane transfer completed;

D: Incubation of strips with antibodies; E: Strip exposure.

p-AKT

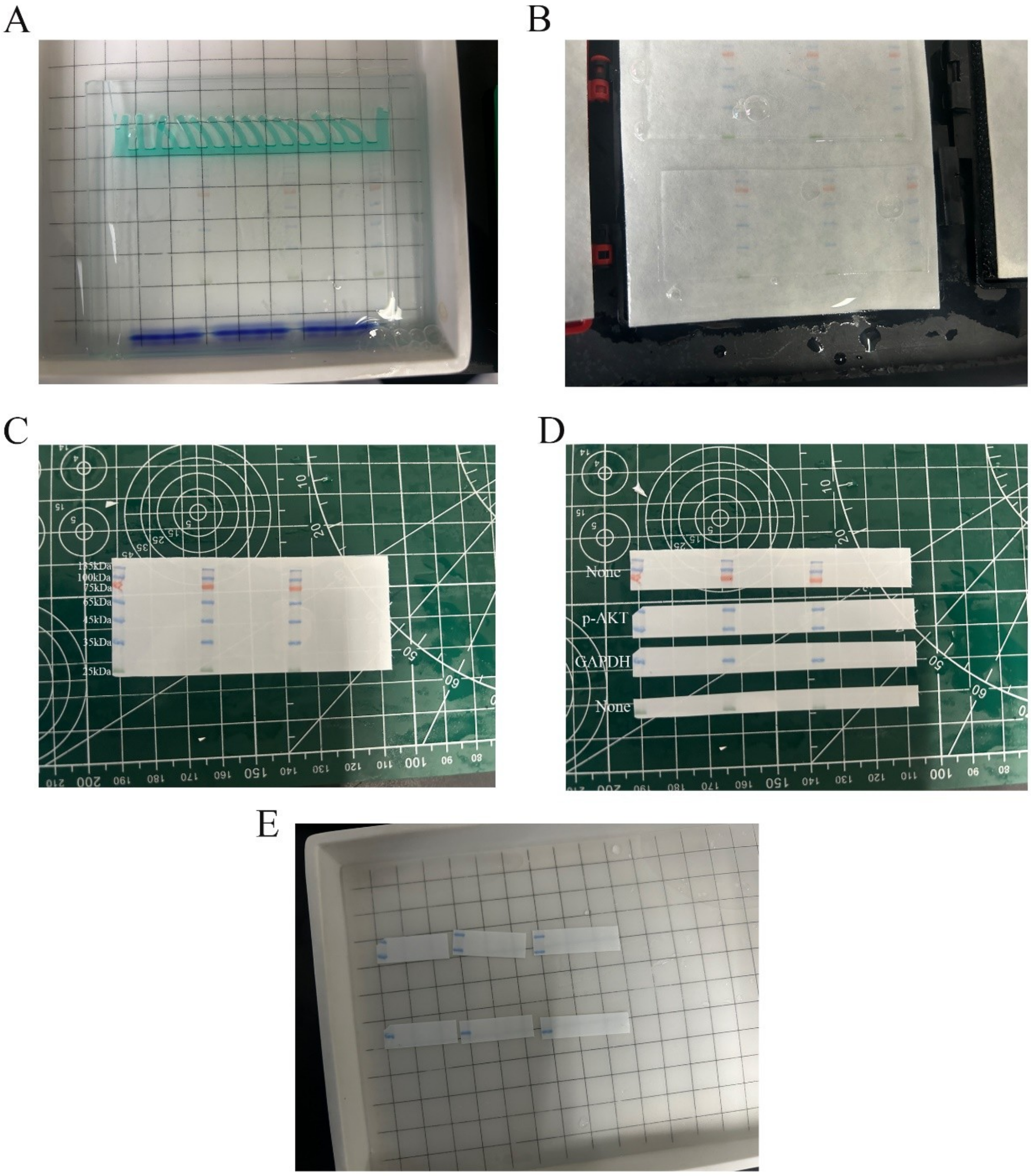

Western blotting flowchart.

A: Gel electrophoresis completed; B: Excess gel removed; C: Membrane transfer completed; D: Incubation of strips with antibodies; E: Strip exposure.

PI3K p110β SOCS3

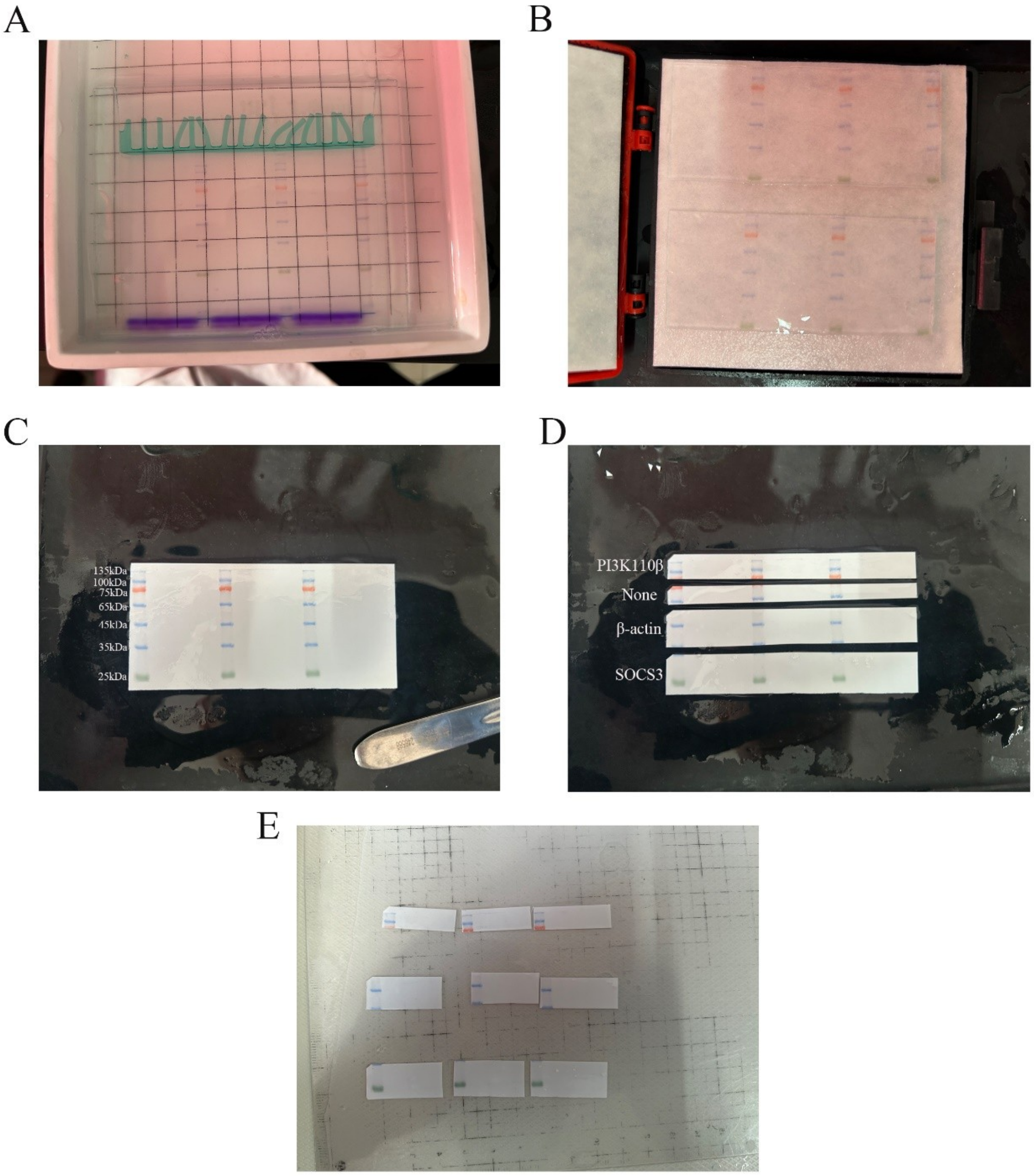

Western blotting flowchart.

A: Gel electrophoresis completed; B: Excess gel removed; C: Membrane transfer completed;

D: Incubation of strips with antibodies; E: Strip exposure.

p-mTOR

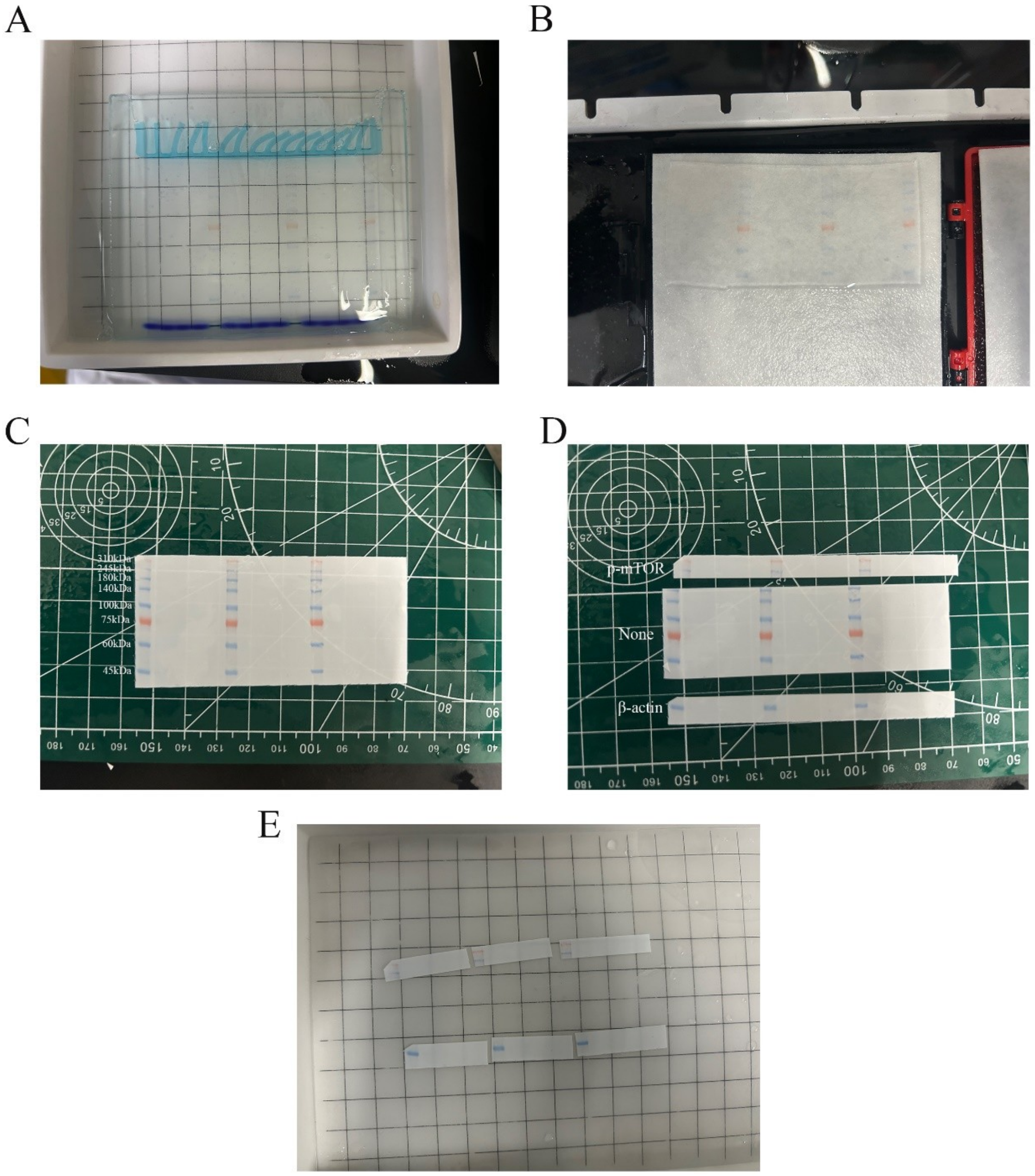

Western blotting flowchart.

A: Gel electrophoresis completed; B: Excess gel removed; C: Membrane transfer completed; D: Incubation of strips with antibodies; E: Strip exposure.

p-PI3Kp110β PCNA

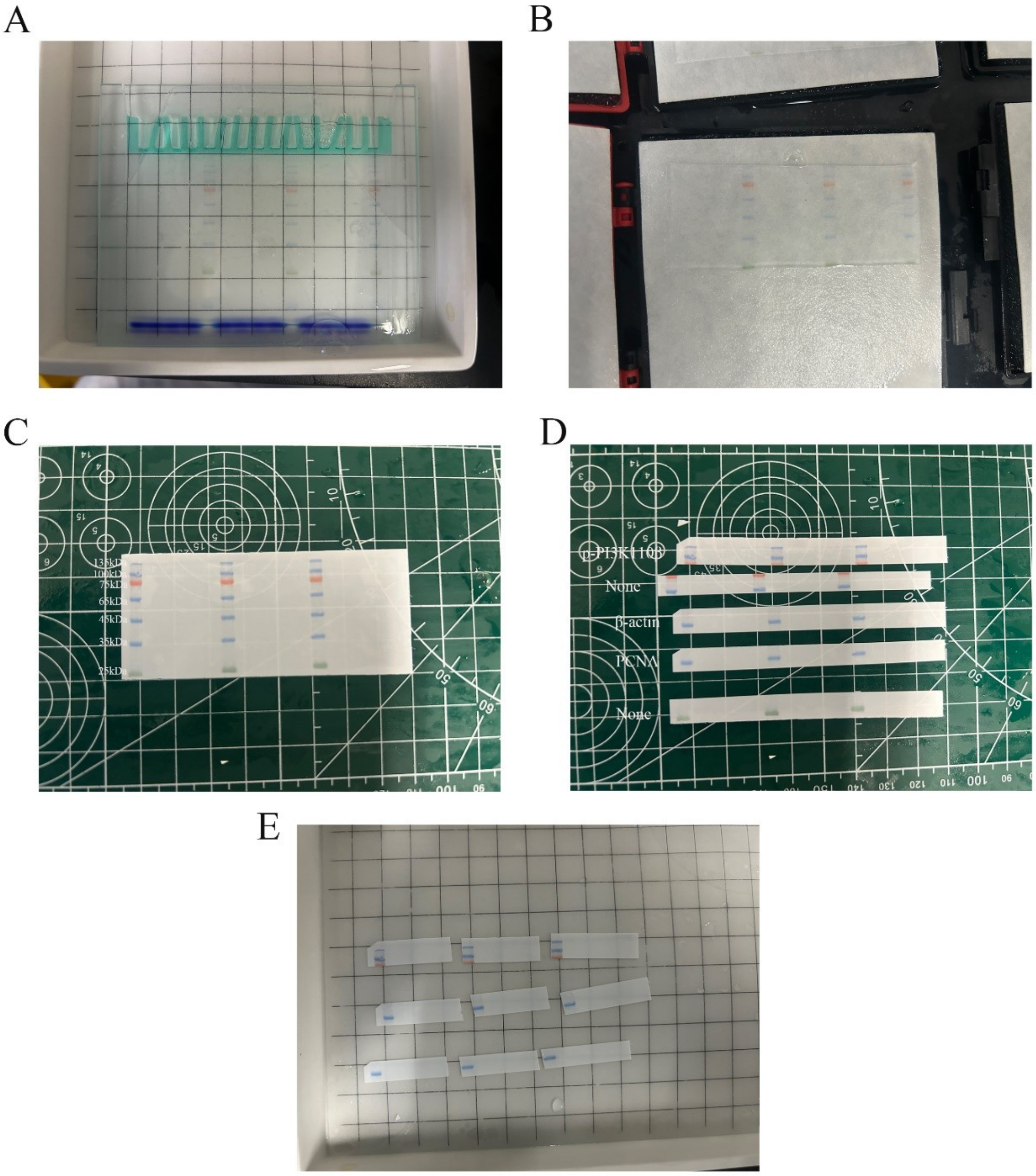

Western blotting flowchart.

A: Gel electrophoresis completed; B: Excess gel removed; C: Membrane transfer completed;

D: Incubation of strips with antibodies; E: Strip exposure.

TGF-β

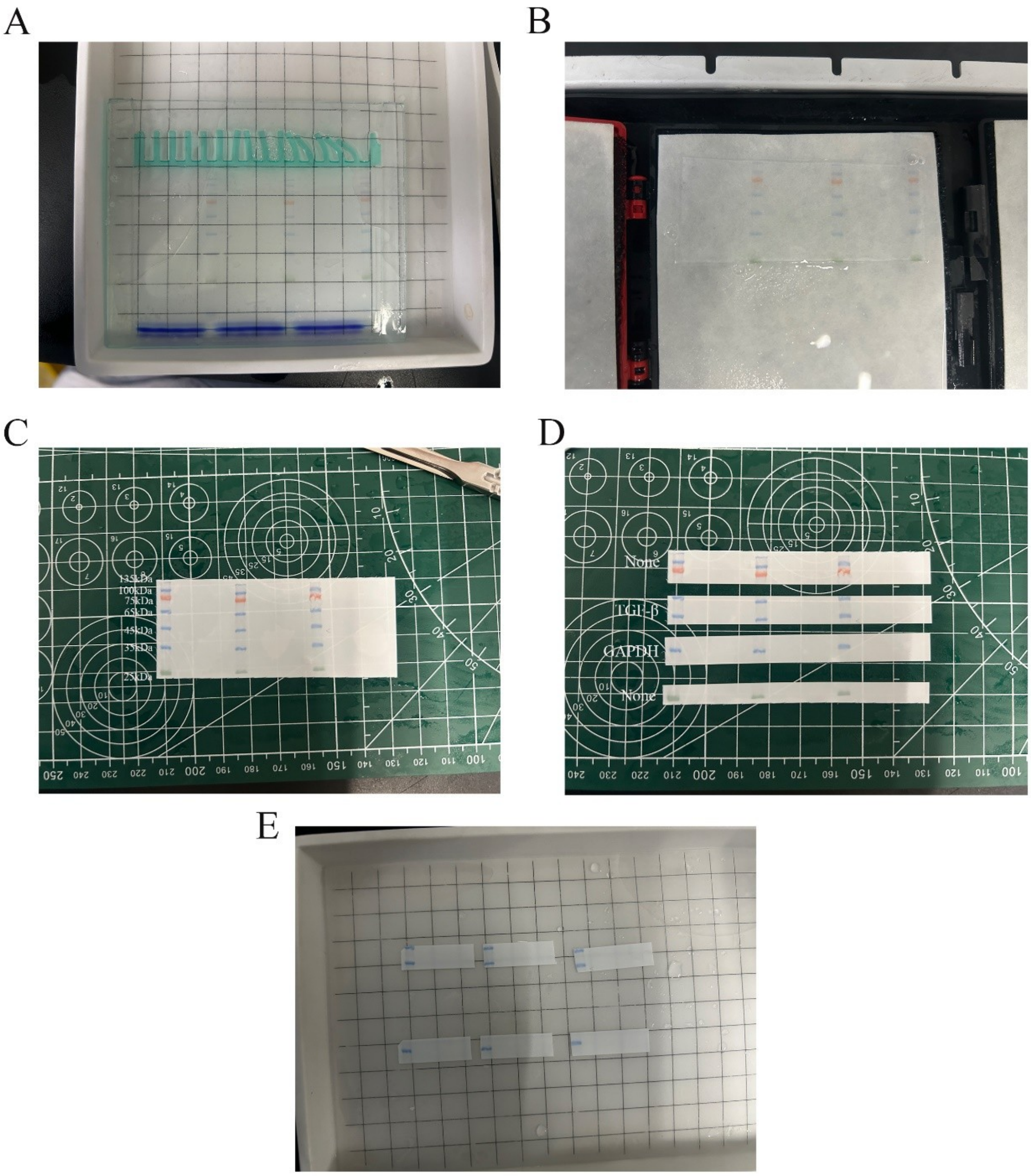

Western blotting flowchart.

A: Gel electrophoresis completed; B: Excess gel removed; C: Membrane transfer completed;

D: Incubation of strips with antibodies; E: Strip exposure.

VEGF

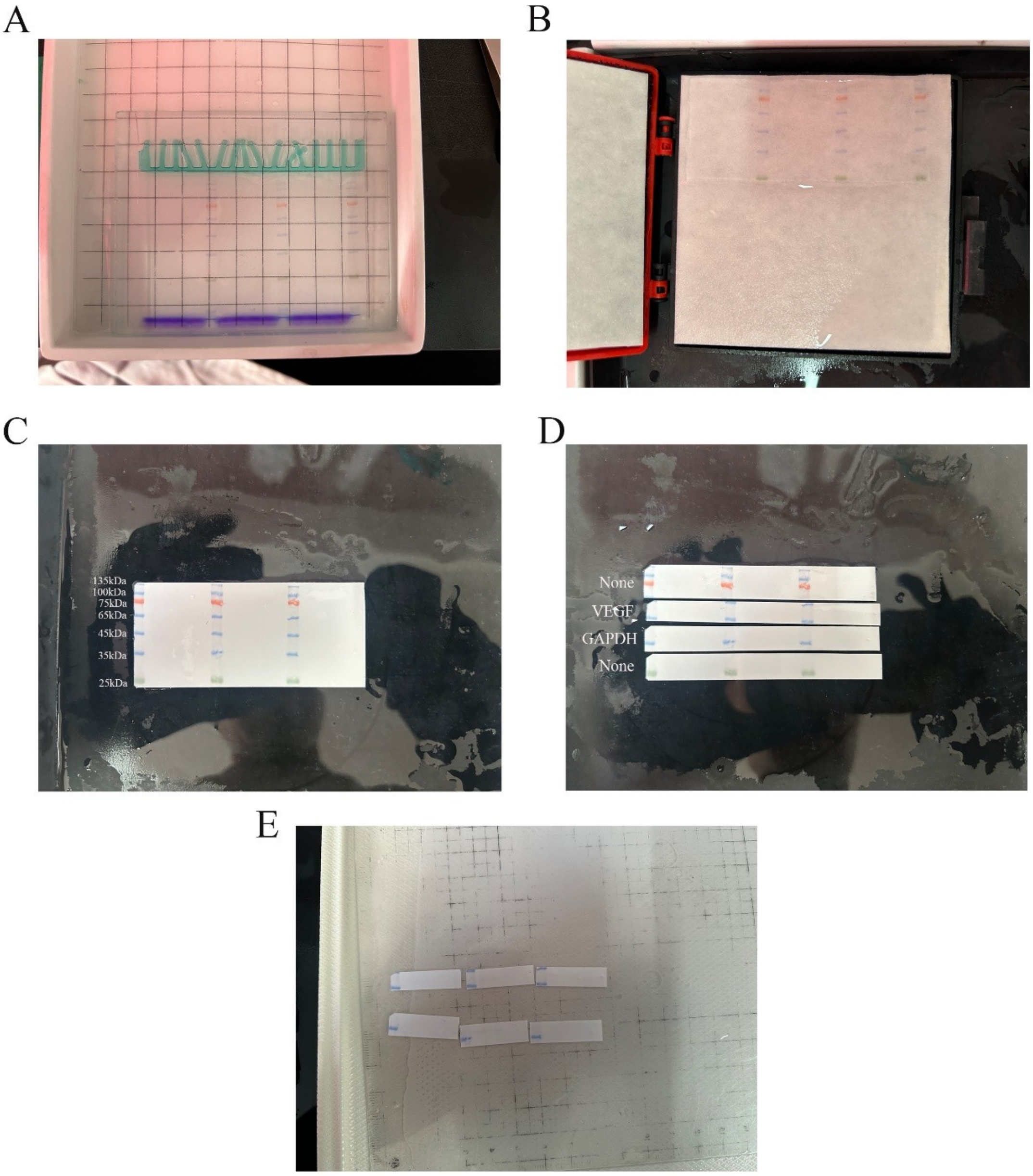

Western blotting flowchart.

A: Gel electrophoresis completed; B: Excess gel removed; C: Membrane transfer completed; D: Incubation of strips with antibodies; E: Strip exposure.
